# *Polygonatum sibiricum* combined with *Poria cocos* prevents spleen deficiency constipation via the gut microbiota–mediated brain–gut axis

**DOI:** 10.3389/fphar.2026.1793441

**Published:** 2026-06-04

**Authors:** Menghui She, Xianqing Sun, Zhoujin Tan

**Affiliations:** 1 School of Traditional Chinese Medicine, Hunan University of Chinese Medicine, Changsha, Hunan, China; 2 School of Pharmacy, Hunan University of Chinese Medicine, Changsha, Hunan, China

**Keywords:** brain-gut peptides, gut microbiota, oxidative stress, *Polygonatum sibiricum*, *Poria cocos*, spleen deficiency constipation

## Abstract

**Introduction:**

The study evaluates the preventive effects of *Polygonatum sibiricum* and *Poria cocos* (3:1) on spleen deficiency constipation in mice, focusing on the brain-gut axis, gut microbiota, and oxidative stress. The findings aim to inform the prevention and treatment of this condition.

**Methods:**

Thirty male specific pathogen-free (SPF) KM mice (4 weeks old, 20 ± 2 g) were randomized into a normal control group (NC), a model group (NM), and a *Polygonatum sibiricum* and *Poria cocos* group (HF). Serum levels of 5-hydroxytryptamine (5-HT), cholecystokinin (CCK), motilin (MTL), and vasoactive intestinal peptide (VIP) were measured by ELISA. Liver superoxide dismutase (SOD) activity and malondialdehyde (MDA) content were measured by a spectrophotometer. Gut microbiota was analyzed by 16S rRNA sequencing.

**Results:**

Compared to NM, the HF showed significantly increased 5-HT, CCK(*p* < 0.05), and MTL (*p* < 0.001), and significantly decreased VIP(*p* < 0.05). Liver MDA content was markedly higher in NM than in NC(*p* < 0.01), with a downward trend in HF. Conversely, the liver SOD activity in NM was significantly lower than in NC(*p* < 0.001), and the HF displayed an upward trend. Microbiota analysis revealed higher diversity in HF, as indicated by higher chao1, pielou_e, shannon, and simpson indices. The gut microbiota species composition in the HF was dominated by beneficial bacteria such as *Bacteroidota* and *Faecalibaculum*, with a reduction in pathogenic bacteria such as *Proteobacteria* and *Escherichia-Shigella*. The characteristic gut microbiota of the NM was enriched in *Escherichia-Shigella* and its conditional pathogens, while the characteristic gut microbiota of the HF was enriched in beneficial symbiotic bacteria such as *Akkermansia*, *Blautia,* and *Bifidobacterium*. The metabolic pathways and the genetic information processing pathway were improved in the HF, and the DNA repair and recombination protein pathways were also repaired.

**Conclusion:**

The *P. sibiricum* and *P. cocos* combination effectively prevents spleen deficiency constipation, an effect associated with the brain-gut axis and gut microbiota. The observed trends in oxidative stress markers warrant further investigation.

## 1 Introduction

Constipation is a pathological condition characterized by difficulty with defecation, prolonged bowel movement cycles (fewer than 3 bowel movements per week), or short cycles with hard, dry stool that is difficult to pass, or non-hard stool with frequent but incomplete bowel movements (Leng et al., 2025). With the acceleration of modern life, increased work pressure, changes in dietary structure, and other factors, the incidence of constipation is becoming increasingly common. According to research, the global prevalence of constipation is approximately 14% (Barberio et al., 2021), while among the elderly population worldwide, it rises to 18.9% (Salari et al., 2023), suggesting an age-related correlation. Additionally, constipation exhibits a gender disparity, with a higher incidence rate in females compared to males (Du et al., 2022). Furthermore, the prevalence of constipation demonstrates an upward trend on an annual basis. If constipation is not effectively treated, it may also lead to a significant increase in the risk of various diseases, such as colorectal cancer, which seriously affects peoples physical and mental health, and the quality of life will also decrease significantly (Scott et al., 2021; Staller et al., 2022). The treatment of constipation in Western medicine typically involves the use of drugs such as dietary fiber supplements, osmotic laxatives, and prokinetic agents. In more severe cases, a colectomy may be necessary (Bharucha and Lacy, 2020). These methods are mainly used to alleviate the symptoms of constipation, but cannot cure it, and can lead to dependency. Rather than waiting to alleviate the condition after it has developed, it is preferable to prevent it before it occurs. Traditional Chinese Medicine (TCM) has always adhered to the theory of “Prevention before Disease Onset, Prevention of Disease from Exacerbating.” Approximately 2000 years ago, the Huangdi Neijing proposed the classic assertion that “sages do not treat diseases that have already appeared, but prevent those that are yet to come.” It indicates that skilled physicians intervene to prevent disease before it manifests, underscoring the importance of prophylaxis.

TCM holds that the primary pathological site for constipation is the large intestine, with a close correlation to the dysfunction of Zangfu organs, such as the lung, spleen, and kidney. It distinguishes between excess and deficiency syndromes, with deficiency syndromes being more common (Fang and Zhu, 2025). This study primarily focuses on spleen deficiency constipation. It should be noted that the spleen referred to in TCM is not equivalent to the spleen in modern anatomy, but rather more akin to a functional complex. The physiological functions of the spleen include dominating transportation and transformation (digesting food and metabolizing fluids), controlling blood (maintaining blood circulation), and dominating sending essence upward (transporting nutrients), which are closely related to the digestive, circulatory, immune, and nervous system in modern medicine (Chen, 2010; Liu et al., 2018). The *qi* in TCM refers to an extremely subtle, vital, and essential substance within the human body. It is the fundamental material that constitutes the body and sustains life activities, as well as the primary driving force of life, possessing functions of promoting, warming, defending, and securing. It is often considered closely related to mitochondria and energy metabolism in modern medical practice (Liu et al., 2020; Lin et al., 2014). Spleen deficiency constipation primarily involves the spleen’s function of governing transportation and transformation. It refers to the process where, after food and drink enter the body, they are transformed into fine substances such as *qi*, blood, and fluids—that is, nutrients in modern medicine—through the action of the spleen, which are then absorbed and transported throughout the Zang fu organs of the body, indicating that the spleen not only aids in digestion and absorption but also regulates fluid metabolism. If the spleen is damaged, its transportation and transformation function is weakened, which will directly affect the digestion and absorption of food and water metabolism, leading to a deficiency in the source of *qi*, blood, and fluids. Insufficiency of *qi* results in a weak transmission capacity of the large intestine, while insufficient body fluids lead to intestinal dryness, thereby causing constipation.

Based on this, the key to preventing spleen deficiency constipation lies in strengthening the spleen and aiding transportation. Both *Polygonatum sibiricum* and *Poria cocos* are food and medicinal herbs. In ancient Chinese medical formulas from various dynasties that contain *P. sibiricum*, it is mentioned that the medicinal pair of *P. sibiricum* and *P. cocos* is used quite frequently (Tao et al., 2021). Additionally, scholars have analyzed the compatibility of patented compound medicines containing *P. sibiricum* and found that the combination of *P. sibiricum* and *P. cocos* ranked third in frequency of occurrence (Ai et al., 2020); indicating that this combination has a wide range of therapeutic effects. According to the Chinese Pharmacopoeia, the recommended therapeutic dosage for both *P. sibiricum* and *P. cocos* is 9–15 g.

Furthermore, our preliminary pre-experiments revealed that neither herb alone is effective for preventing constipation. *Polygonatum sibiricum* is characterized as sweet and neutral in nature, entering the lung, spleen, and kidney meridians, with the effects of tonifying the spleen and nourishing the kidney, supplementing *qi* and nourishing *yin* (Chinese Pharmacopoeia Commission, 2020). On one hand, its effect of supplementing *qi* can promote intestinal peristalsis and enhance its conduction capacity; on the other hand, its efficacy of nourishing *yin* can moisten the intestines and alleviate the condition of constipation, which is highly consistent with the pathogenesis of spleen deficiency constipation. Consequently, we increased the dosage of *P. sibiricum* to serve as the main herb for prevention. Nevertheless, the solitary or excessive use of tonifying herbs like *P. sibiricum* can lead to a greasy, cloying nature. This heaviness can, in turn, obstruct the spleen, thereby impairing its functions of transformation and transportation. To counteract this, a small amount of *P. cocos* was added to strengthen the spleen and drain dampness (Chinese Pharmacopoeia Commission, 2020). We found that a 3:1 ratio of *P. sibiricum* to *P. cocos* demonstrates a certain efficacy in preventing spleen deficiency constipation. Accordingly, this study further investigates the mechanism of action of the 3:1 ratio, aiming to achieve more effective prevention of spleen deficiency constipation.

Modern research indicates that *P. sibiricum* and *P. cocos* are also clinically used to treat intestinal diseases. The main chemical components of Polygonatum sibiricum are polysaccharides, saponins, and flavonoids, which possess pharmacological effects such as lowering blood sugar and lipid levels, regulating the immune system, and exhibiting antioxidant activity (Liu et al., 2024; Ren et al., 2024). Researchers have found that *P. sibiricum* can regulate the activity of the gut microbiota (Liu et al., 2022); *P. sibiricum* polysaccharides can improve intestinal function, such as restoring tissue structure, alleviating oxidative stress, reducing intestinal permeability, and balancing the composition of the gut microbiota (Li et al., 2022); Polygonatum saponins can improve intestinal barrier damage by targeting the PI3K/AKT/mTOR-mediated autophagy/microbiota axis (Xiao et al., 2024). The chemical constituents of *P. cocos* include polysaccharides, triterpenoids, amino acids, lecithin, and other substances (Lei et al., 2025), and possess pharmacological effects such as diuretic, anti-inflammatory, immune-enhancing, and antioxidant activities (Guo ZY. et al., 2025; Xie et al., 2025; Lu et al., 2021). Researchers have found that *P. cocos* and its extracts can regulate gastrointestinal motility, thereby enhancing digestive function and fluid metabolism in rats with spleen deficiency (Feng et al., 2023). *Poria cocos* polysaccharides can restore the gut microbiota homeostasis in rats with spleen deficiency, effectively improving their immune function (Zhang et al., 2024). Previous research has primarily focused on the individual effects of *P. sibiricum* and *P. cocos*, leaving a gap in understanding their combined application. To date, no study has examined the preventive effects of a *P. sibiricum* and *P. cocos* combination in a 3:1 ratio for spleen deficiency constipation. Moreover, existing studies have focused on treating constipation after it has developed, whereas our research centers on preventive intervention before its onset.

Moreover, research indicates a strong association among constipation, brain‐gut peptides, oxidative stress, and gut microbiota. Dysregulation of brain-gut peptides can impair intestinal motility, thereby triggering constipation (Dou et al., 2017). Key gut-brain peptide molecules include serotonin (5-HT), cholecystokinin (CCK), motilin (MTL), and vasoactive intestinal peptide (VIP). Typically, reduced levels of 5-HT, CCK, and MTL signify slowed gastrointestinal peristalsis, which hinders the expulsion of intestinal contents and contributes to constipation. Conversely, VIP acts as an inhibitory neurotransmitter; elevated VIP levels suppress gastrointestinal motility, thereby provoking constipation (Wang P. et al., 2024). In addition, heightened oxidative stress causes free radical damage to effector cells, leading to cellular degeneration, intestinal mucosal damage, and constipation (Jiang et al., 2024). This constipation, in turn, irritates the intestinal mucosa, triggering an inflammatory response that generates more oxygen free radicals and exacerbates peroxidative damage (Yi et al., 2023a). MDA is a common biomarker for assessing oxidative stress (Tsaturyan et al., 2022), while SOD functions as an antioxidant that helps mitigate oxidative stress (Anwar et al., 2025). Additional studies have established that gut microbiota dysbiosis is a primary factor in the onset of constipation (Wang et al., 2022).

In summary, the study is designed to investigate the association between the preventive efficacy of the *P. sibiricum* and *P. cocos* combination (3:1) in a mouse model of spleen deficiency constipation, and to examine changes in brain-gut peptides, gut microbiota, and oxidative stress biomarkers. The findings are intended to provide novel insights for the prevention of spleen deficiency constipation, establish a foundation for future mechanistic research, and advance the use of *P. sibiricum* and *P. cocos* within the food and pharmaceutical sectors.

## 2 Materials and methods

### 2.1 Experimental animals and breeding environment

Male specific pathogen-free (SPF) grade Kunming (KM) mice, 4 weeks of age and weighing (20 ± 2) g, were acquired from Hunan SJA Laboratory Animal Co., Ltd [License number: SCXK (Xiang) 2019–0004]. The mice were housed in the Experimental Animal Center of Hunan University of Chinese Medicine [License No: SYXK (Xiang) 2019–0009], where they were provided with standard rodent chow. The environment was maintained at 23 °C–25 °C, 50%–70% humidity, and a 12-h light/dark cycle, with free access to food and water. This study was approved by the Animal Ethics Committee of Hunan University of Chinese Medicine and was conducted in accordance with ethical guidelines for animal research [Ethics No: SLBH-202505150004].

### 2.2 Experimental drugs and preparation

*Polygonatum sibiricum* and *P. cocos* decoction: The equivalent daily dose for mice was calculated based on body surface area conversion, resulting in 1.95 g/(kg⋅d) administered twice daily at 0.35 mL per dose. The final concentration of the solution was 0.098 g/mL. *Polygonatum sibiricum* from Xinhua County and *P. cocos* from Jingzhou County were ground and mixed at a 3:1 ratio (15 g *P. sibiricum* and 5 g *P. cocos*). The mixture was soaked in 205 mL of water for 30 min, then decocted for an additional 30 min. After cooling, the solution was stored at 4 °C.

*Folium Sennae* decoction: The equivalent daily dose for mice was calculated to be 20 g/(kg⋅d) administered once daily at 0.35 mL per dose, yielding a concentration of 1 g/mL. A 100 g sample of *Folium Sennae* was soaked in water for 30 min, then decocted with five times its volume of water for 30 min. The resulting liquid was filtered through gauze. The remaining plant material was decocted again with an additional volume of water for 15 min, then filtered. The two filtrates were combined and concentrated to a final concentration of 1 g/mL using a rotary evaporator at 60 °C and 15 rpm/min. The final decoction was stored at 4 °C.

### 2.3 Grouping and dosage regimen

Following a 3-day acclimatization period, 30 KM mice were randomly assigned to three groups of ten each using a random number table: normal control group (NC), model group (NM), and *P. sibiricum* and *P. cocos* group (HF).

For 14 days prior to the experiment, the HF received the *P. sibiricum* and *P. cocos* decoction by gavage (0.35 mL, twice daily). The NC and NM received an equal volume of sterile water on the same schedule. During the subsequent 15-day experimental phase, the NM and HF underwent induction of constipation using the spleen deficiency model. This involved 7 days of *Folium Sennae* decoction gavage followed by 8 days of dietary and water restriction. Throughout the modeling period, the HF continued to receive its assigned solution to assess a preventative effect, while the NC remained on a normal diet and received sterile water.

### 2.4 Animal model preparation

The animal model of spleen deficiency constipation was established using a combination of methods adapted from the research group’s previous work and those of other scholars (Yi et al., 2023b; Wu et al., 2023; Liang et al., 2025; Ao and Zhang, 2024). The composite modeling approach involved gavage with *Folium Sennae* decoction, irregular feeding patterns, restricted water intake, and a low-fiber diet. The 15-day modeling process was conducted in two phases. The spleen deficiency phase (Days 1–7): Mice received daily gavage of *Folium Sennae* decoction (0.35 mL) while maintainig *ad libitum* access to food and water to induce the spleen deficiency state. The constipation phase (Days 8–15): *Folium Sennae* gavage was discontinued. Mice were subjected to irregular feeding and water restriction, receiving a low-fiber diet (4–8 g of raw rice per mouse) and only 0.5 h of free access to water daily for 8 days to induce constipation on the established basis of spleen deficiency.

Senna is a cold-natured medicine whose coldness can damage the spleen and stomach; irregular eating habits can also lead to spleen deficiency. Furthermore, methods such as water restriction and a low-fiber diet can induce constipation, indicating that the establishment of this model is consistent with the pathogenic factors of human spleen deficiency constipation. In addition, mice in NM showed manifestations of spleen deficiency and dry stools-symptoms that are consistent with those of human spleen deficiency constipation-indicating that the method of establishing this model aligns with the research framework for TCM disease and syndrome models (You et al., 2020).

### 2.5 Model evaluation

The successful induction of spleen deficiency constipation model was confirmed through a multidimensional assessment of the mice. It included observations of physical appearance (withered, thin, piloerection, arched back), reduced locomotor activity, decreased body weight, and abnormal fecal characteristics (dry, reduced in quantity, and small pellet size). Post-mortem examination revealed fecal impaction in the colon, forming bead-like or ball-like masses, with no significant fecal residue in the jejunum or ileum. This comprehensive evaluation of outward appearance, behavior, defecation patterns, anatomical findings, and biomarkers confirmed that the induced model accurately reflected the pathological features of spleen deficiency constipation.

Spleen deficiency affects digestive function, manifesting as reduced gastrointestinal motility, intestinal barrier damage, and gut microbiota imbalance (Yi et al., 2022; Guo K. et al., 2025). Constipation is defined as difficulty in defecation. Furthermore, spleen deficiency constipation also affects oxidative stress and brain-gut peptide levels (Zou et al., 2009). Therefore, the primary manifestations of spleen deficiency constipation in mice include impaired digestive function and changes in fecal characteristics, such as dry, shriveled, and small appearance. Systemic symptoms include piloerection, kyphosis, reduced activity, and weight loss. This is because spleen’s inability to digest and absorb nutrients leads to insufficient nutritional supply, resulting in symptoms of malnutrition such as emaciation. Upon dissection, fecal matter is found to be aggregated in the colon, appearing spherical or bead-like, with no significant fecal residue in the jejunum and ileum (Zhu et al., 2022).

### 2.6 Main reagents

Mouse Serotonin (5-HT) ELISA Research Kit (Cat# JM-02726M2), Mouse Cholecystokinin (CCK) ELISA Research Kit (Cat# JM-02311M2), Mouse Motilin (MTL) ELISA Research Kit (Cat# JM-02775M2), Mouse Vasoactive Intestinal Peptide (VIP) ELISA Research Kit (Cat# JM-02729M2), the aforementioned kits were procured from Jiangsu Jingmei Biotechnology Co., Ltd. Superoxide Dismutase (SOD) Activity Assay Kit (BC0175), Malondialdehyde (MDA) Content Assay kit (BC0025), the aforementioned kits were procured from Beijing Solarbio Science & Technology Co., Ltd.

### 2.7 Experimental instruments

LC-RE-301 Rotary Evaporator (Shanghai Lichenbangxi Instrument Technology Co., Ltd.); UV-Vis Spectrophotometer (Nano drop 2000/2000C, Thermo Scientific, USA); Vortex Mixer (VORTEX 5, Kylin-Bell Instruments); Multifunctional Microplate Reader (Model SPARK, Tecan Trading AG, Switzerland); High-Speed Refrigerated Centrifuge (Model 5810R, Eppendorf, Germany); Autoclave (Model SQ510C, Yamato Scientific Co., Ltd.); Electric Blast Drying Oven (GZX-9070MBE, Shanghai Boxing Industrial Co., Ltd. Medical Equipment Factory); Electric Thermostatic Incubator (Model 303-4B, Shaoxing Shangcheng Instrument Manufacturing Co., Ltd.); Electronic Balance (YP302N, Shanghai Jinghai Instrument Co., Ltd.); Water Bath Pot (SSW-420, Shanghai Boxing Industrial Co., Ltd.).

### 2.8 Indicator detection

#### 2.8.1 General condition of mice

During the modeling and administration period, the general condition of the mice, including mental state, activity level, reactivity, and fur condition, was observed daily. On the final day, the fecal water content was measured.

$$\text{Fecal water content }\left(\%\right)=\left(\text{Fecal wet weight}-\text{Fecal dry weight}\right)/\text{Fecal wet weight}\times 100\%$$

#### 2.8.2 Serum ELISA assay

Following inhalation anesthesia with isoflurane, blood was collected via the retro-orbital plexus, and the mice were then euthanized by cervical dislocation. The blood samples were allowed to clot at room temperature for approximately 3 h before the serum was collected by centrifugation at 3,000 rpm/min for 10 min. Serum levels of serotonin (5-HT), cholecystokinin (CCK), vasoactive intestinal peptide (VIP), and motilin (MTL) were determined by enzyme-linked immunosorbent assay (ELISA) according to the manufacturer instructions.

#### 2.8.3 Determination of liver MDA content and SOD activity

From the mouse livers obtained via dissection as described in Section 2.8.2, five mice per group were randomly selected. Two 0.1 g liver tissue samples were separately homogenized in 1 mL of MDA and SOD kit extraction solution on ice, followed by centrifugation at 8,000 *g* and 4 °C for 10 min. The MDA content and SOD activity in the liver homogenate were measured by a spectrophotometer, with procedures performed in strict accordance with the manufacturers protocol.

#### 2.8.4 Hematoxylin and eosin (HE) staining of colon tissue

A 1 cm segment of the colon was aseptically collected and fixed in 4% paraformaldehyde. The tissue was then processed through dehydration, clearing, paraffin embedding, sectioning, and staining with hematoxylin and eosin. Histopathological examination of the colon tissue was performed using an optical microscope and CaseViewer software.

#### 2.8.5 16 S rRNA high-throughput sequencing

1. Sample collection: Under aseptic conditions, colonic contents were extruded using forceps. The intestinal tissue was then longitudinally incised with sterile scissors, and residual colonic contents were flushed with physiological saline. The intestinal wall tissue was recovered, excess moisture was removed with filter paper, and colonic mucosal tissue was scraped and collected using sterile cover slips. The mucosal tissue from each mouse was placed in a cryogenic vial and stored at −80 °C. Samples were sent to Novogene Bioinformatics Technology Co., Ltd. For second-generation high-throughput sequencing to analyze gut microbiota structure and diversity using the 16 S rRNA gene.
2. Sequencing data processing and evaluation: Raw sequence data were processed through modification, trimming, filtering, denoising, and merging steps, followed by DADA2 analysis to generate high-quality, valid sequences. Data quality was assessed using dilution curves, species accumulation curves, and the coverage variation index.
3. Species annotation: Sequence data were analyzed using QIIME2. Sequences were clustered at 100% similarity to generate Amplicon Sequence Variants (ASVs) and an abundance table. The Greengenes database was used to assign taxonomic information to each ASV by matching its sequence against reference sequences.
4. Alpha diversity analysis: Alpha diversity characterizes species richness, diversity, and evenness. The Chao1, Observed species, Shannon, and Simpson indices were calculated from the ASV table in QIIME2 to assess these metrics.
5. Beta diversity analysis: Beta diversity quantifies differences in species composition between samples. The Bray-Curtis dissimilarity metric was used to analyze beta diversity and investigate structural variations in the microbial community. Principal Coordinates Analysis (PCoA) and Nonmetric Multidimensional Scaling (NMDS) were employed for visualizing these differences.
6. Species composition analysis: Composition and abundance tables for each sample were generated at the phylum and genus levels based on ASV clustering and taxonomic identification. Bar charts were used to visualize and compare the relative abundances of different taxonomic groups across samples.
7. Characteristic microbiota analysis: Linear Discriminant Analysis Effect Size (LEfSe) was employed to identify taxonomic units that were significantly enriched between different groups. This method visualizes the biomarker species within each group and their hierarchical taxonomic distribution.
8. Functional prediction analysis: The metabolic potential of the gut microbiota was predicted using PICRUSt2 to infer functional units. These units were mapped against the Kyoto Encyclopedia of Genes and Genomes (KEGG) database to predict the abundance of various metabolic pathways.
9. Correlation analysis: A co-occurrence network of species at the phylum level was constructed using Spearman correlation. Redundancy Analysis (RDA) was utilized to examine the interactive effects of the *P. sibiricum* and *P. cocos* compound on the gut microbiota and environmental factors in a mouse model of spleen deficiency constipation.

### 2.9 Statistical analysis

Data processing and statistical analysis were performed using SPSS 25.0 software. Graphical representations were created with GraphPad Prism 9 and other software. Data are presented as the mean ± standard deviation ( $\bar{x}\pm s$ ). For comparisons among multiple groups, a one-way ANOVA followed by the LSD *post hoc* test was used when data were normally distributed and variances were homogeneous. The Kruskal–Wallis H test was used for data that did not meet these assumptions. Statistical significance was defined at α = 0.05 (Significance levels: *p < 0.05, **p < 0.01, ***p < 0.001).

## 3 Results

### 3.1 Effects of the combination of *Polygonatum sibiricum* and *Poria cocos* on the general condition of mice

**FIGURE 1:**
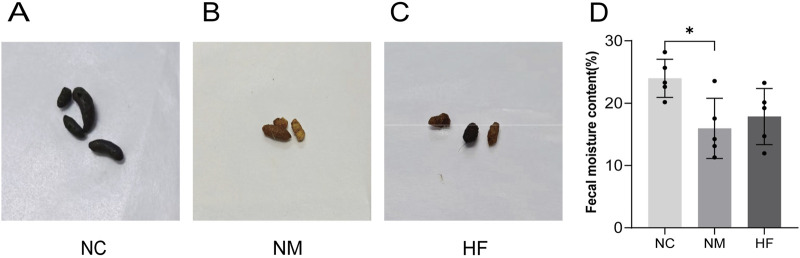
General physiological signs in mice. **(A)** fecal morphology from the NC; **(B)** fecal morphology from the NM; **(C)** fecal morphology from the HF; **(D)** fecal moisture content (%) for all groups. Note: NC, normal control group; NM: model group; HF, *Polygonatum sibiricum* and *Poria cocos* group.

Following the experiment, the mice in the NC exhibited a healthy mental state, high activity levels, responsiveness, and glossy fur. In contrast, the mice in the NM showed poor mental status, reduced activity, a tendency to huddle in corners, dry fur, and a generally thinner physique. Mice in the HF group demonstrated a robust mental state, restored fur luster, and heightened responsiveness, closely resembling those in the NC. As illustrated in Figure 1, the feces of the NC were black and moderately firm (Figure 1A). Feces from the NM were yellow, composed of smaller, harder particles that were difficult to crush (Figure 1B). Feces from the HF were brown with a slightly soft texture, closer to the NC (Figure 1C).

Furthermore, as shown in Figure 1D, the fecal moisture content of the NM was significantly lower (*p* < 0.05), and the fecal moisture content of the HF of mice showed an increasing trend, but this difference did not reach statistical significance.

### 3.2 Effects of the combination of *Polygonatum sibiricum* and *Poria cocos* on colon pathology in mice with spleen deficiency constipation

**FIGURE 2:**
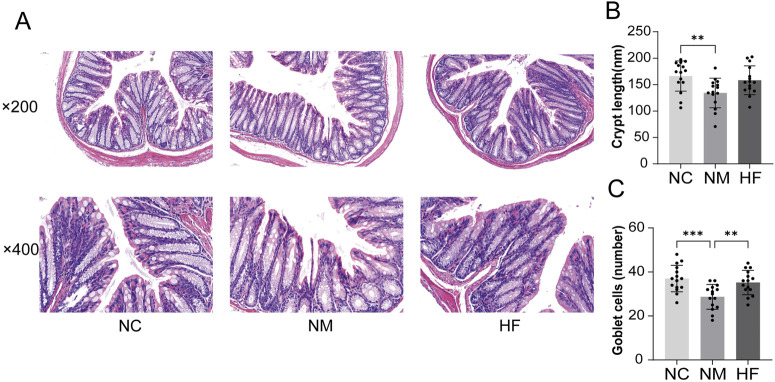
Pathological morphology of the colon in mice. **(A)** Histological sections of colon tissue from each group; **(B)** Colon crypt length; **(C)** The number of colon goblet cells. Note: NC, normal control group; NM, model group; HF, *Polygonatum sibiricum* and *Poria cocos* group.

A normal intestine is composed of the mucosal, submucosal, muscular, and serosal layers. As shown in Figure 2A, the colon tissue of the NC displayed a clear and intact structure with no observable edema, inflammation, or lymphocyte infiltration in the mucosal layer. In comparison to the NC, the NMs colon tissue structure remained clear but exhibited disrupted mucosal continuity, scattered lymphocyte infiltration accompanied by edema, and a thinning of the muscularis mucosae. The colon tissue in the HF showed significantly reduced mucosal damage, with a clear, complete structure and no signs of edema, inflammation, or lymphocyte infiltration.

Quantitative analysis showed that, compared with the NM, the HF crypt length showed a trend of recovery, but this difference did not reach statistical significance (Figure 2B). In addition, the number of goblet cells in the HF was significantly increased (*p* < 0.01) (Figure 2C), indicating that *P. sibiricum* and *P. cocos* effectively alleviated intestinal barrier damage induced by constipation.

### 3.3 Effects of the combined administration of *Polygonatum sibiricum* and *Poria cocos* on serum levels of 5-HT, CCK, MTL, and VIP in mice with spleen deficiency constipation

**FIGURE 3:**
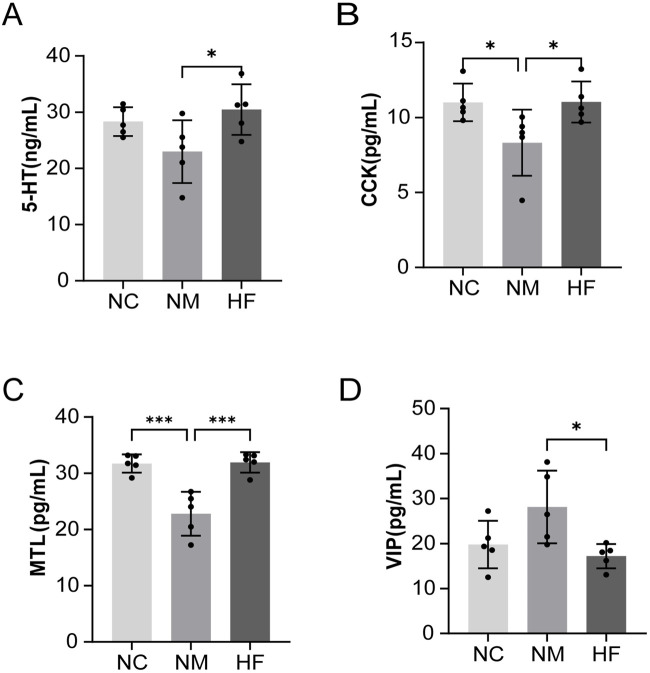
Serum levels of 5-HT, CCK, VIP, and MTL in mice from each group. **(A)** 5-HT content; **(B)** CCK content; **(C)** MTL content; **(D)** VIP content. Note: NC: normal control group; NM: model group; HF: *Polygonatum sibiricum* and *Poria cocos* group.

Brain-gut peptides are small molecules distributed in the brain and gut that can regulate gastrointestinal motility (Jiang et al., 2020). This class of peptides includes 5-HT, CCK, MTL, and VIP. As shown in Figure 3A, the serum 5-HT concentration in the model group did not differ significantly from that in the normal group, but showed a decreasing trend. In contrast, the HF exhibited a significantly elevated serum 5-HT level compared with the NM (*p* < 0.05). Regarding serum CCK levels (Figure 3B), the NM showed a significant reduction compared to the NC (*p* < 0.05), a deficit that was significantly reversed by the HF (*p* < 0.05). Similarly, for serum MTL (Figure 3C), the NMs level was profoundly lower than that of the NC (*p* < 0.001), and the HF demonstrated a highly significant increase compared to the NM (*p* < 0.001). For serum VIP (Figure 3D), no significant difference was found between the NM and NC, though an upward trend was noted. Conversely, the HF showed a significant decrease in serum VIP compared with the model group (*p* < 0.05). Thus, brain-gut peptide levels in the NM were significantly altered, whereas those in the HF approached normal values. It suggests that the combination of *P. sibiricum* and *P. cocos* may help restore brain-gut peptide levels and improve gastrointestinal motility.

### 3.4 Effects of the combined administration of *Polygonatum sibiricum* and *Poria cocos* on liver MDA content and SOD activity in mice with spleen deficiency constipation

**FIGURE 4:**
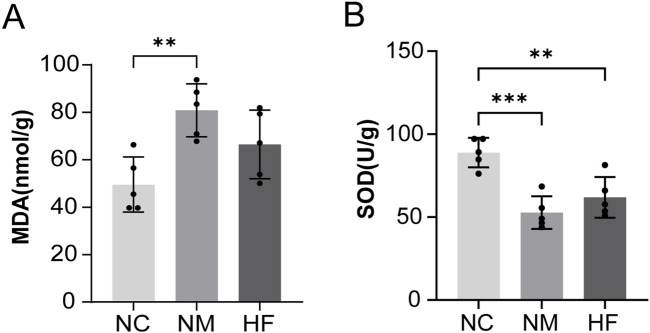
Liver MDA content and SOD activity in mice. **(A)** MDA content; **(B)** SOD activity. Note: NC, normal control group; NM, model group; HF, *Polygonatum sibiricum* and *Poria cocos* group.

In the liver, the MDA content of mice in the NM was markedly elevated compared to the NC (*p* < 0.01). Although the MDA content in the HF showed a decreasing trend compared with the NM, this difference was not statistically significant (Figure 4A). Conversely, SOD activity was significantly reduced in the NM (*p* < 0.001). While the HF showed a tendency towards increased SOD activity, this change did not attain statistical significance (Figure 4B).

### 3.5 Effects of the combined administration of *Polygonatum sibiricum* and *Poria cocos* on the gut microbiota in mice with spleen deficiency constipation

#### 3.5.1 Effect of *Polygonatum sibiricum* and *Poria cocos* on the diversity of intestinal mucosal microbiota in mice

**FIGURE 5:**
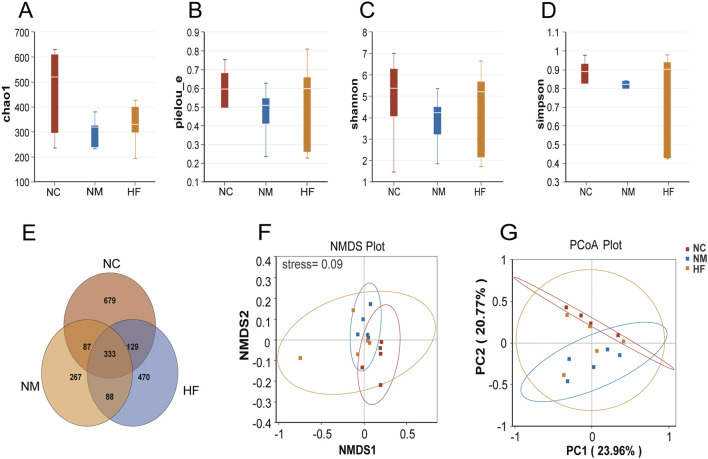
Analysis of microbial diversity in the colonic contents of mice. **(A)** The chao1 index; **(B)** the pielou_e index; **(C)** the shannon index; **(D)** the simpson index **(E)** the Venn diagram; **(F)** the NMDS analysis; **(G)** the PCoA analysis. Note: NC, normal control group; NM, model group; HF, *Polygonatum sibiricum* and *Poria cocos* group.

As shown in Figure 5E, the NC comprised 1,187 ASVs, of which 679 were unique. The NM comprised 817 ASVs, including 267 unique ASVs. The HF comprised 1,019 ASVs, including 470 unique ASVs. There were 87 shared ASVs between the NC and NM, 129 between the NC and HF, 88 between the NM and HF, and 333 across all three groups. These results indicate a reduction in the total number of ASVs, accompanied by a decrease in the number of unique ASVs. These results indicate a reduction in the total number of ASVs, accompanied by a decrease in the number of unique ASVs.The higher ASV count in the NC and HF compared to the lowest count in the NM suggests that HF can restore intestinal microbial diversity in mice with spleen deficiency constipation.

For Alpha diversity, we assessed the chao1, pielou_e, shannon, and simpson indices. Compared to the NM, the HF exhibited upward trends in all four indices (Figures 5A–D), although these differences were not statistically significant. Beta diversity analysis, which compared microbial community composition across different samples, used NMDS to capture the datas nonlinear structure effectively. Figure 5F shows distinct structural distributions of communities on the colonic mucosa of mice in each group, with a stress value of 0.09, confirming the validity of the groupings. In the PCoA analysis (Figure 5G), PC1 accounted for 23.96% of the variance, and PC2 accounted for 20.77%. Both PCoA and NMDS analyses revealed clustering within each group, with substantial dissimilarity in community structure between the NC and NM and partial overlap between the HF and the other two groups. These findings demonstrate that the gut microbiota composition within the colonic mucosa is markedly altered in mice with spleen deficiency constipation. Furthermore, *P. sibiricum* and *P. cocos* may exert their effects on the body by modulating the structure of the gut microbiota.

#### 3.5.2 Effect of *Polygonatum sibiricum* and *Poria cocos* on the species composition of the intestinal microbiota in mice

**FIGURE 6:**
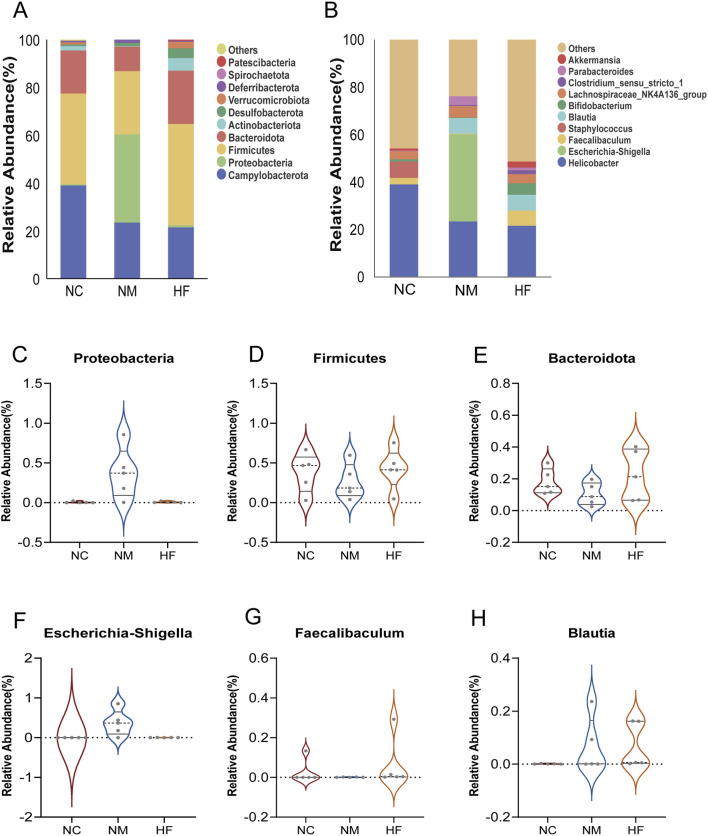
Analysis of the species composition of gut microbiota in mice. **(A)** The relative abundance chart at the phylum level; **(B)** the relative abundance chart at the genus level; **(C)** the dominant phylum Proteobacteria; **(D)** the dominant phylum Firmicutes; **(E)** the dominant phylum *Bacteroidota*; **(F)** the dominant genus Escherichia-Shigella; **(G)** the dominant genus *Faecalibaculum*; **(H)** the dominant genus Blautia. Note: NC: normal control group; NM: model group; HF: *Polygonatum sibiricum* and *Poria cocos* group.

We further statistically analyzed the relative abundance of the top 10 dominant bacterial phyla and genera. As depicted in Figure 6A, the dominant phyla colonizing the colonic mucosa of the experimental mice in all three groups were primarily *Campylobacterota*, *Firmicutes*, and *Bacteroidota*. Moreover, compared with the NC, we observed a significant increase in *Proteobacteria* abundance in the NM (Figure 6C), whereas the HF showed a profile similar to that of the NC. Furthermore, the relative abundances of both *Firmicutes* and *Bacteroidota* were diminished in the NM relative to both the NC and HF (Figures 6D,E). At the genus level (Figure 6B), *Helicobacter*, *Escherichia-Shigella*, *Faecalibaculum*, and *Staphylococcus* were identified as the predominant genera.

In comparison to the NC, the NM exhibited increased abundances of *Escherichia-Shigella* and *Blautia* (Figures 6F,H), and a decreased abundance of *Faecalibaculum* (Figure 6G). Conversely, the HF demonstrated the opposite trend, with abundances approaching those of the NC. It suggests that the preventive effect of *P. sibiricum* and *P. cocos* on spleen deficiency constipation may be associated with modulating the abundance of dominant phyla and genera.

#### 3.5.3 Effect of the *Polygonatum sibiricum* and *Poria cocos* combination on the characteristic mucosal bacteria in mice

**FIGURE 7:**
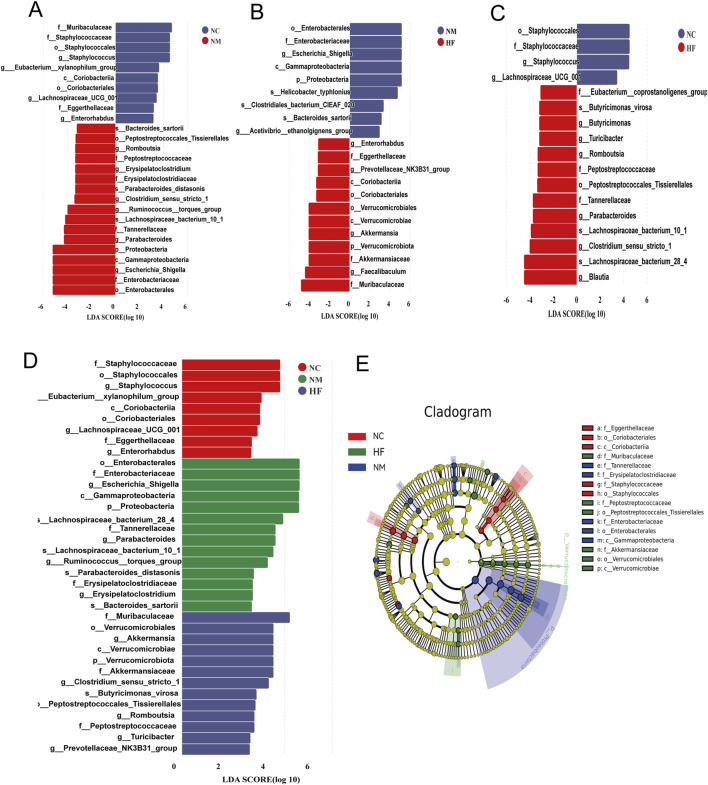
Analysis of the characteristic flora in the intestinal mucosa of mice from each group. **(A)** The bar chart showing the LDA score distribution between the NC and NM; **(B)** is the bar chart showing the LDA score distribution between the NM and HF; **(C)** The bar chart showing the LDA score distribution between the NC and HF; **(D)** The bar chart showing the LDA score distribution across all three groups; **(E)** LEfSe cladogram. Note: NC: normal control group; NM: model group; HF: *Polygonatum sibiricum* and *Poria cocos* group.

LEfSe is an analytical tool for discovering and interpreting high-dimensional biomarkers, emphasizing statistical significance and biological relevance to identify biomarkers with significant differences between groups. We set the Linear Discriminant Analysis (LDA) score threshold at >3 for the comparison. Figure 7A displays the characteristic bacteria for the comparison between the NC and NM groups. The NC was primarily characterized by *Muribaculaceae*, *Staphylococcaceae*, and *Staphylococcales*, while the NM was characterized by *Escherichia-Shigella*, *Proteobacteria*, *Gammaproteobacteria*, and *Enterobacteriaceae*. Comparing the NM and HF, the NM group was characterized by *Enterobacterales*, *Enterobacteriaceae*, *Escherichia-Shigella*, *Gammaproteobacteria*, and *Proteobacteria*. In contrast, the HF group was characterized by *Muribaculaceae*, *Faecalibaculum*, and *Akkermansia* (Figure 7B). In the comparison between the NC and HF groups, the NC group was characterized by *Staphylococcales*, *Staphylococcaceae*, and *Staphylococcus*, while the HF group was enriched in *Blautia*, *Bifidobacterium*, *Bifidobacteriales*, and *Actinobacteria* (Figure 7C). A comparison among all three groups further confirmed that the NC was characterized by *Staphylococcaceae*, *Staphylococcales*, *Staphylococcus*, and *Coriobacteriales*; the NM was characterized by *Enterobacterales*, *Enterobacteriaceae*, and *Escherichia-Shigella*; whereas the HF was characterized by *Muribaculaceae*, *Verrucomicrobiales*, and *Akkermansia* (Figure 7D). The LEfSe cladogram revealed that the NC group had branches enriched for *Eggerthellaceae*, *Coriobacteriales*, and *Staphylococcaceae*. The NM group showed enrichment in branches related to *Gammaproteobacteria*, *Enterobacteriaceae*, and *Tannerellaceae*. The HF group exhibited enrichment in branches such as *Verrucomicrobiae*, *Akkermansiaceae*, *Peptostreptoccaceae*, and *Muribaculaceae*, with an overall taxonomic distribution more similar to that of the NC group (Figure 7E). The results revealed distinct characteristic bacterial profiles among the three groups. The NM showed enrichment of *Escherichia-Shigella* and other opportunistic pathogenic bacteria, whereas the HF showed enrichment of beneficial commensal bacteria, including *Akkermansia*, *Blautia*, and *Bifidobacterium*, which are conducive to maintaining intestinal homeostasis.

#### 3.5.4 Effects of the *Polygonatum sibiricum* and *Poria cocos* combination on the intestinal flora function in mice

**FIGURE 8:**
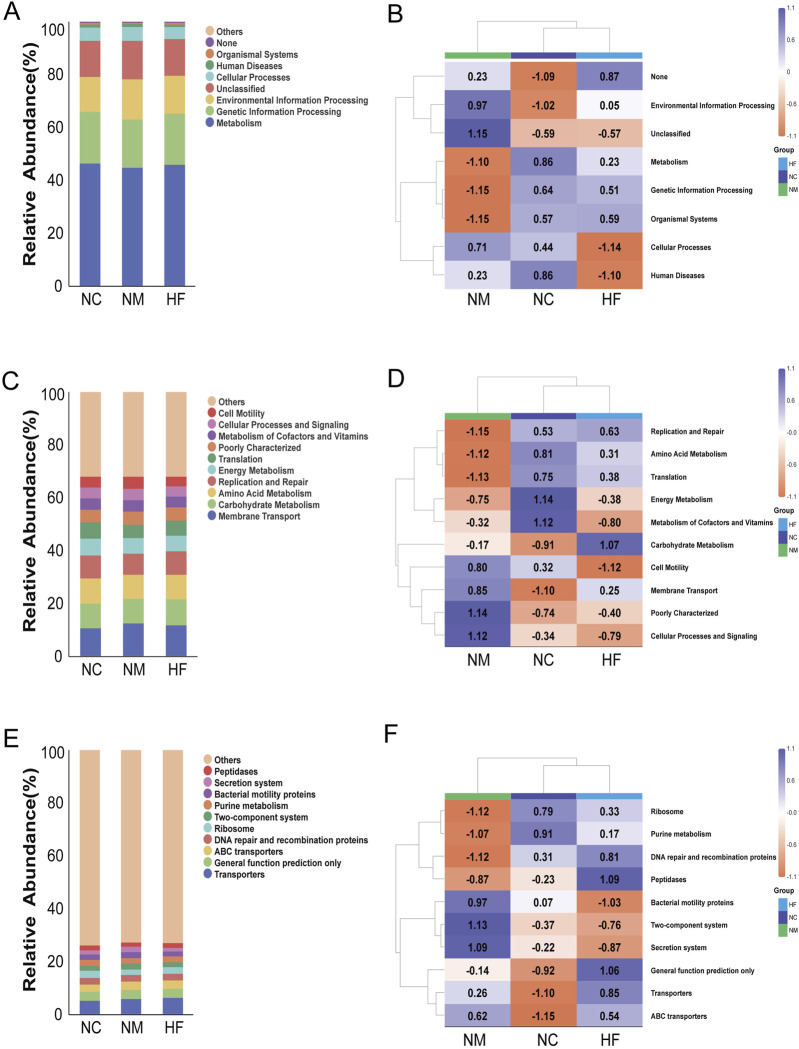
Functional analysis of the intestinal flora in mice. **(A)** The bar chart of relative abundance at Level 1; **(B)** The cluster heatmap of relative abundance at Level 1; **(C)** The bar chart of relative abundance at Level 2; **(D)** The cluster heatmap of relative abundance at Level 2; **(E)** The bar chart of relative abundance at Level 3; **(F)** The cluster heatmap of relative abundance at Level 3. Note: NC: normal control group; NM: model group; HF: *Polygonatum sibiricum* and *Poria cocos* group.

Using PICRUSt analysis, we selected the 10 pathways with the highest relative abundance at each level for functional prediction. The Level 1 analysis indicated that the overall functional abundance was dominated by *Metabolism*, *Genetic Information Processing*, and *Environmental Information Processing* (Figure 8A). Specifically, the NC exhibited higher abundance in the *Metabolism* and *Genetic Information Processing* pathways, whereas the NM showed higher abundance in the *Environmental Information Processing* and *Cellular Processes* pathways. The HF demonstrated higher abundance in *Organismal Systems* and *Genetic Information Processing* pathways (Figure 8B). The Level 2 analysis revealed that the overall functional abundance was primarily concentrated in *Membrane Transport*, *Carbohydrate Metabolism*, and *Amino Acid Metabolism* (Figure 8C). The NC had higher abundance in *Energy Metabolism*, *Metabolism of Cofactors and Vitamins*, and *Translation* pathways. The NM displayed higher abundance in *Poorly Characterized* and *Cellular Processes* and *Signaling pathways*, whereas the HF showed higher abundance in *Translation and Replication* and *Repair* pathways, a pattern more similar to the normal control group (Figure 8D). At the Level 3 analysis, the overall functional abundance was mainly *Transporters*, *General function prediction only*, and *ABC transporters* (Figure 8E). The NC had higher abundance in *Purine Metabolism*, *Ribosome*, and *DNA Repair and Recombination Proteins* pathways. The NM exhibited higher abundance in the *Two-Component System*, *Secretion System*, and *Bacterial Motility Proteins* pathways. In contrast, the HF showed higher abundance in *Peptidases* and *DNA Repair and Recombination Proteins* pathways, a profile closer to that of the NC (Figure 8F).

These findings suggest that metabolic and genetic information-processing pathways are impaired in mice with spleen deficiency constipation, and that intestinal flora function may shift towards environmental information processing, cellular processes, signaling, and bacterial motility proteins. *Polygonatum sibiricum* and *P. cocos* may prevent spleen deficiency constipation by modulating metabolic pathways.

#### 3.5.5 Correlation analysis

**FIGURE 9:**
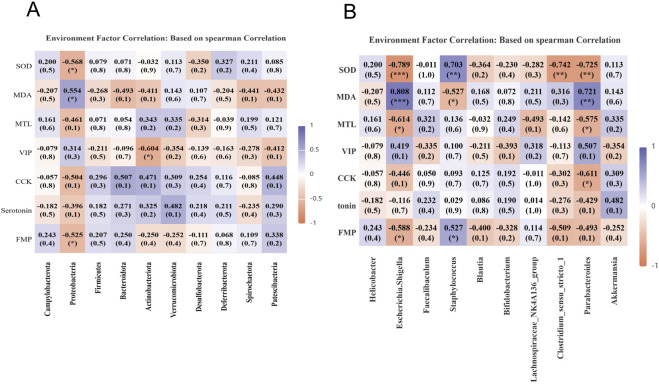
Correlation analysis between the intestinal microbiota and environmental factors in mice. **(A)** The correlation analysis of environmental factors at the phylum level; **(B)** The correlation analysis of environmental factors at the genus level. Note: NC, normal control group; NM, model group; HF, *Polygonatum sibiricum* and *Poria cocos* group.

Using Spearman correlation analysis, we selected the top 10 most abundant species to investigate the relationships between environmental factors associated with spleen deficiency constipation and the intestinal microbiome. At the phylum level (Figure 9A), SOD showed a positive correlation with *Patescibacteria*, *Firmicutes*, and *Bacteroidota*, and a significant negative correlation with *Proteobacteria* (*p* < 0.05). MDA demonstrated a significant positive correlation with *Proteobacteria* (*p* < 0.05) and negative correlations with *Bacteroidota* and *Patescibacteria*. MTL was negatively correlated with *Proteobacteria* and positively correlated with *Actinobacteriota* and *Verrucomicrobiota*. VIP showed a significant negative correlation with *Actinobacteriota* (*p* < 0.05) and a positive correlation with *Proteobacteria*. CCK was positively correlated with *Bacteroidota* and *Actinobacteriota* and negatively correlated with *Proteobacteria*. 5-HT was positively correlated with *Verrucomicrobiota* and negatively correlated with *Proteobacteria*. FMP was positively correlated with *Patescibacteria* and significantly negatively correlated with *Proteobacteria* (*p* < 0.05). At the genus level (Figure 9B), SOD was significantly positively correlated with *Staphylococcus* (*p* < 0.01) and significantly negatively correlated with *Escherichia-Shigella* (*p* < 0.001) and *Parabacteroides* (*p* < 0.01). MDA was significantly positively correlated with *Escherichia-Shigella* (*p* < 0.001) and *Parabacteroides* (*p* < 0.01) and significantly negatively correlated with *Staphylococcus* (*p* < 0.05). MTL was positively correlated with *Akkermansia* and significantly negatively correlated with *Escherichia-Shigella* and *Parabacteroides* (*p* < 0.05). VIP was positively correlated with *Parabacteroides* and *Escherichia-Shigella* and negatively correlated with *Bifidobacterium* and *Akkermansia*. CCK was positively correlated with *Akkermansia* and significantly negatively correlated with *Parabacteroides* (*p* < 0.05). 5-HT was positively correlated with *Akkermansia* and negatively correlated with *Parabacteroides*. FMP was significantly positively correlated with *Staphylococcus* (*p* < 0.05) and significantly negatively correlated with Escherichia-Shigella (*p* < 0.05). These correlations suggest that the antioxidant SOD is positively associated with beneficial phyla, such as *Bacteroidota*. At the same time, the oxidative stress marker MDA is positively correlated with pathogenic bacteria in *Proteobacteria* and *Escherichia-Shigella*, a pattern mirrored by VIP, MTL, CCK, and 5-HT, which are positively correlated with beneficial phyla such as *Verrucomicrobiota*, *Actinobacteriota*, and *Bacteroidota*, as well as the beneficial genus *Akkermansia*. It suggests that the dysbiosis observed in the NM may result from the enrichment of opportunistic pathogens due to elevated levels of MDA and VIP, or a reduction in beneficial bacteria caused by decreased levels of SOD, MTL, CCK, and 5-HT. *Polygonatum sibiricum* and *P. cocos* may positively improve oxidative stress and brain-gut peptide levels, inhibit the growth of harmful bacteria, modulate the microbial community structure, and thereby maintain intestinal health.

## 4 Discussion

### 4.1 The combination of *Polygonatum sibiricum* and *Poria cocos* prevents spleen deficiency constipation in mice by modulating brain-gut peptides to influence the brain-gut axis

Gut-brain peptides are small peptide molecules secreted by endocrine cells in the gastrointestinal tract, constituting the fundamental substance for the “brain-gut axis.” In physiological states, these peptides regulate gastrointestinal motility via the central nervous system and gastrointestinal smooth muscle cells (Tang et al., 2021). Dysregulation of their expression can lead to delayed gastric emptying and diminished intestinal peristalsis, ultimately resulting in constipation (Niu et al., 2023). Key gut-brain peptide molecules include 5-HT, CCK, MTL, and VIP (Reich and Hölscher, 2024), which function as both neurotransmitters and hormones, playing a pivotal role in modulating gastrointestinal motility and nutrient absorption (Wu et al., 2022). This research demonstrates that the HF exhibited significant elevations in 5-HT, CCK, and MTL concentrations. Specifically, 5-HT, a crucial neurotransmitter, is associated not only with cognitive and emotional processes but also with intestinal motility and defecation. Increased 5-HT levels generally indicate enhanced gastrointestinal peristalsis, which promotes the expulsion of intestinal contents (Sharp and Barnes, 2020; Wen, 2025). CCK, which can modulate neuronal activity (Kamalova et al., 2024), has been shown to stimulate gastrointestinal smooth muscle contraction and regulate motility (Shi et al., 2021). MTL accelerates gastric emptying and augments colonic contractions (Yang and Yang, 2025); heightened MTL levels are therefore beneficial for alleviating constipation. Moreover, the HF showed a significant reduction in VIP, an inhibitory neurotransmitter. A decrease in VIP secretion alleviates suppression of gastrointestinal smooth muscle, thereby boosting intestinal motility and increasing glandular secretions, which, in turn, relieves constipation (Gao et al., 2025).

A profound interconnection exists between the gut and the brain (Quigley, 2017), with the gut microbiota acting as a central regulator of this brain-gut interaction. The microbiota influences intestinal function and, through the enteric nervous system, can affect neural processes in the brain (Li et al., 2019; Evrensel et al., 2019). Studies confirm that gut microbes can modulate neurotransmitter levels (Strandwitz, 2018) and influence the production of gut-brain peptides, thereby mediating functions along the brain-gut axis (Khan et al., 2025). These findings are strongly consistent with the results of our study. Correlation analysis further revealed that MTL, CCK, and 5-HT were positively correlated with beneficial microbial populations, such as *Verrucomicrobiota*, *Bacteroidota*, and *Akkermansia*, whereas VIP showed a positive correlation with opportunistic pathogens, such as *Proteobacteria* and *Escherichia-Shigella*. We hypothesize that *P. sibiricum* and *P. cocos* may regulate the gut microbiota to influence the secretion of brain-gut peptides, thereby maintaining homeostasis of the brain-gut axis and preventing spleen deficiency constipation.

### 4.2 The combination of *Polygonatum sibiricum* and *Poria cocos* prevents spleen deficiency constipation in mice by modulating the gut microbiota

The integrity of the intestinal barrier forms the structural foundation for maintaining microbial homeostasis. The histopathology findings from this study demonstrate that *P. sibiricum* and *P. cocos* increase the number of goblet cells and repair the intestinal barrier, thereby creating a favorable microenvironment for intestinal microbial activity. The gut microbiota plays a pivotal role in human health, influencing physiological processes such as nutrient metabolism and immune regulation (Zhang et al., 2020). Our previous work has established that gut microbiota dysbiosis can trigger constipation (Yi et al., 2023b). This study revealed that, compared with the NM, the HF exhibited greater mucosal flora diversity, suggesting its potential to correct microbial imbalances.

Linear discriminant analysis (LEfSe) further highlighted the differences in characteristic flora between the groups. The NMs characteristic flora was enriched with opportunistic pathogens, such as *Escherichia-Shigella*, which are known to induce inflammation and serve as biomarkers of intestinal inflammation (Hong et al., 2024; Wang C. et al., 2024). Conversely, the characteristic flora in the HF was enriched with beneficial symbionts such as *Akkermansia*, *Blautia*, and *Bifidobacterium*. Specifically, *Akkermansia* enhances antioxidant capacity and aids intestinal barrier repair (Zhang et al., 2025; Ghaffari et al., 2023)^;^ *Blautia* contributes to colonic mucus formation and provides protective effects on the gut microbiota (Holmberg et al., 2024); and *Bifidobacterium* modulates the gut microbiota, improves barrier function, and reduces inflammatory markers (Wierzbicka et al., 2021). Clinically, composite probiotics containing *Bifidobacterium* and *Lactobacillus* are frequently used to manage constipation symptoms (Luo et al., 2025; He et al., 2022), supporting the therapeutic value of microbiota modulation for this condition. It indicates that *P. sibiricum* and *P. cocos* may function as prebiotics, selectively fostering the growth of beneficial bacteria while suppressing pathogenic bacteria to preserve intestinal health and stability.

In terms of functional prediction, the model group mice showed diminished activity in metabolic and genetic information processing pathways, likely due to impaired nutrient absorption and intestinal epithelial cell damage during constipation. However, the HF not only restored these pathways but also bolstered the activity of DNA repair and recombinant protein pathways. It suggests that *P. sibiricum* and *P. cocos* may enhance the gut epithelium ability to repair oxidative and other forms of damage by modulating the microbiota, thereby breaking the “dysbiosis-barrier damage” cycle. Future studies should investigate these pathways in conjunction with metabolomics and transcriptomics to elucidate the molecular mechanisms by which *P. sibiricum* and *P. cocos* regulate spleen deficiency constipation.

### 4.3 The combination of *Polygonatum sibiricum* and *Poria cocos* prevents spleen deficiency constipation in mice by modulating oxidative stress

Prior research by our group identified oxidative stress as a potential key mechanism in mouse constipation, findings consistent with those of this study. The NM exhibited a marked elevation in MDA content and a significant reduction in SOD activity. As a lipid peroxidation metabolite, MDA serves as an indicator of cellular damage caused by oxygen-free radicals; higher MDA levels correlate with more severe cellular injury (Muro et al., 2024). SOD, an antioxidant enzyme, effectively scavenges oxygen-free radicals produced during cellular metabolism, maintaining intracellular redox homeostasis and mitigating oxidative stress (You et al., 2020).

We observed a decrease in MDA content and an increase in SOD activity in the HF. While these changes were not statistically significant, their directional trend aligns with the established antioxidant effects of Chinese herbal medicine. This lack of statistical significance could be attributable to the limited duration of the intervention or an inadequate sample size. As the intervention time is prolonged or the sample size is increased, the antioxidant effect of *P. sibiricum* and *P. cocos* may become significant. Future relevant research will require more diverse samples to confirm the relevant conclusions. In addition, correlation analysis showed that SOD activity was significantly negatively correlated with the abundance of opportunistic pathogens such as *Proteobacteria* and *Escherichia-Shigella*, while MDA content was positively correlated with them. It indicates that improvements in oxidative stress status are closely related to reductions in the abundance of these opportunistic pathogens. In terms of gut microbiota composition, the microbiota in the NM was characterized by enrichment of genera such as *Escherichia-Shigella* and *Proteobacteria*, which are positively correlated with oxidative stress. After the intervention with *P. sibiricum* and *P. cocos*, not only was the enrichment of the aforementioned opportunistic pathogens reduced, but the growth of beneficial symbiotic bacteria, such as *Akkermansia*, *Blautia*, and *Bifidobacterium,* was also promoted. It is known that *Proteobacteria*, including the genera *Escherichia-Shigella*, are closely related to oxidative stress levels. Their abundance changes are often regarded as biological indicators of an oxidative stress environment.

We speculate that the *P. sibiricum* and *P. cocos* combination may mitigate the hosts oxidative stress, in part, by restructuring the gut microbiota. It involves suppressing opportunistic pathogens that are positively correlated with oxidative stress (such as *Proteobacteria*, *Escherichia-Shigella*) and enriching beneficial commensal bacteria. Consequently, targeted intervention on the gut microbiota could be a viable approach when oxidative stress is present.

In summary, we speculate that the combination of *P. sibiricum* and *P. cocos* can prevent spleen deficiency constipation by regulating gut microbiota, thereby influencing brain-gut peptide levels and oxidative stress. We acknowledge several limitations inherent to this study. First, the data establish correlations, not causality. We did not conduct the multiple causal pathway validations necessary to establish definitive links. Specifically, we did not perform experiments to remove microbial communities to see if the effects are dependent on gut bacteria; we did not use fecal microbiota transplantation (FMT) to test if changing the microbiota is enough to create a protective effect; nor did we use specific inhibition of receptors or signaling pathways to find a direct mechanism. Thus, while our results suggest potential mechanisms, they do not prove a causal relationship. Second, Oxidative stress was assessed exclusively in liver tissue, rather than in the intestine, which is most pertinent to constipation. Moreover, the alterations in these markers were not statistically significant, merely indicating a non-significant trend. Consequently, the contribution of oxidative stress to the observed preventive effects remains hypothetical. Further research is required to definitively establish this role, which should involve measuring intestinal-specific markers of oxidative stress, such as reactive oxygen species (ROS) levels, glutathione, or 8-hydroxydeoxyguanosine (8-OHdG) in colonic tissue.

Nevertheless, our findings provide a solid foundation for future multicenter studies. The spleen deficiency constipation model used in this study is more consistent with the etiology and pathogenesis of Traditional Chinese Medicine, thereby better reflecting its traditional clinical manifestations. Regarding intervention timing, prior research has commonly employed herbal treatments only after the onset of constipation. Furthermore, by consolidating the analysis of brain-gut peptides, gut microbiota, and oxidative stress into a single experimental framework, we enabled a multifactorial analysis, which lends significant credibility to our conclusions.

## 5 Conclusion

In summary, the study demonstrates that the *P. sibiricum*: *P. cocos* (3:1) combination is effective in preventing spleen deficiency constipation. This efficacy is associated with alterations in brain-gut peptide levels, shifts in the gut microbiota composition, and changes in oxidative stress biomarkers. These findings elucidate potential mechanisms underlying the preventive effects and lay the foundation for future mechanistic research using causal inference methods. Moreover, the study supports the industrial application of *P. sibiricum* and *P. cocos* in the food and pharmaceutical sectors, thereby improving the utilization of medicinal herbs and fostering the development of new therapeutic, nutraceutical, and functional food products for disease prevention and treatment.

## Data Availability

The datasets presented in this study can be found in online repositories. The names of the repository/repositories and accession number(s) can be found at: https://www.ncbi.nlm.nih.gov/bioproject/PRJNA1292445/.

## References

[B1] Ai H. P. Wen Y. Q. Zuo Y. L. Li G. (2020). Analysis of clinical application and formula compatibility of patented compound prescriptions containing polygonatum based on data mining. Med. Innovation China 17 (6), 141–144.

[B2] Anwar S. Sarwar T. Khan A. A. Rahmani A. H. (2025). Therapeutic applications and mechanisms of superoxide dismutase (SOD) in different pathogenesis. Biomolecules 15, 1130. 10.3390/biom15081130 40867576 PMC12384489

[B3] Ao X. Zhang Z. (2024). Effect of xylo-oligosaccharides on intestinal bacterial diversity in mice with spleen deficiency constipation. Front. Microbiol. 15, 1474374. 10.3389/fmicb.2024.1474374 39473853 PMC11519740

[B4] Barberio B. Judge C. Savarino E. V. Ford A. C. (2021). Global prevalence of functional constipation according to the rome criteria: a systematic review and meta-analysis. Lancet Gastroenterol. Hepatol. 6, 638–648. 10.1016/S2468-1253(21)00111-4 34090581

[B5] Bharucha A. E. Lacy B. E. (2020). Mechanisms, evaluation, and management of chronic constipation. Gastroenterology 158 (5), 1232–1249.e3. 10.1053/j.gastro.2019.12.034 31945360 PMC7573977

[B6] Chen S. M. (2010). Advances in the modern study of the physiological functions of the spleen in traditional Chinese medicine over the last twenty years. J. Basic Chin. Med. 16 (04), 348–351. 10.19945/j.cnki.issn.1006-3250.2010.04.039

[B7] Chinese Pharmacopoeia Commission (2020). Pharmacopoeia of the Peoples Republic of China (Volume I). Beijing: China Medical Science Press, 319–320.

[B8] Dou Y. C. Xu Q. Q. Meng X. Y. (2017). Research progress of brain gut peptides, anxiety and depression in constipation. Chin. J. Gastroenterol. Hepatol. 26 (5), 497–502. 10.3969/j.issn.1006-5709.2017.05.006

[B9] Du X. Liu S. Jia P. Wang X. Gan J. Hu W. (2022). Epidemiology of constipation in elderly people in parts of China: a multicenter study. Front. Public Health 10, 823987. 10.3389/fpubh.2022.823987 35784241 PMC9240593

[B10] Evrensel A. Önen Ünsalver B. Ceylan M. E. (2019). Therapeutic potential of the microbiome in the treatment of neuropsychiatric disorders. Med. Sci. (Basel) 7, 21. 10.3390/medsci7020021 30709065 PMC6410187

[B11] Fang G. D. Zhu J. H. (2025). Analysis on idea of all five zang-organs leading to occurrence of constipation from syndrome differentiation of zang-fu organs in huangdi neijing. China J. Tradit. Chin. Med. Pharm. 40 (3), 1353–1356.

[B12] Feng M. Jia Z. M. Wan M. Fan B. L. Tang X. Q. Chen W. H. (2023). Effectiveness of fuling (poria) and its extracts against spleen deficiency in rats *via* tonifying spleen. J. Tradit. Chin. Med. 43 (3), 501–506. 10.19852/j.cnki.jtcm.2023.03.002 37147751 PMC10133940

[B13] Gao Z. Y. Zhou X. L. Xi Z. W. Dong F. Y. Wu Q. Cao D. (2025). Effects of baizhu tongmi prescription on intestinal neurotransmitter factors in elderly patients with slow transit constipation of qi-stagnation syndrome, based on the “brain-gut axis” theory. Chin. J. Gerontol. 45 (17), 4152–4155.

[B14] Ghaffari S. Abbasi A. Somi M. H. Moaddab S. Y. Nikniaz L. Kafil H. S. (2023). Akkermansia muciniphila: from its critical role in human health to strategies for promoting its abundance in human gut microbiome. Crit. Rev. Food Sci. Nutr. 63, 7357–7377. 10.1080/10408398.2022.2045894 35238258

[B15] Guo Z. Y. Wu X. Zhang S. J. Yang J. h. Miao H. Zhao Y. y. (2025a). Poria cocos: traditional uses, triterpenoid components and their renoprotective pharmacology. Acta Pharmacol. Sin. 46 (4), 836–851. 10.1038/s41401-024-01404-7 39482471 PMC11950336

[B16] Guo K. Tang Y. Yang T. Yan Y. W. (2025b). Massa medicata fermentata treated spleen deficiency constipation by mediating intestinal microbiota and serum peptide. Front. Cell. Infect. Microbiol. 15, 1556915. 10.3389/fcimb.2025.1556915 40115071 PMC11923552

[B17] He Y. Zhu L. Chen J. Tang X. Pan M. Yuan W. (2022). Efficacy of probiotic compounds in relieving constipation and their colonization in gut microbiota. Molecules 27 (3), 666. 10.3390/molecules27030666 35163930 PMC8838973

[B18] Holmberg S. M. Feeney R. H. Prasoodanan P. K. V. Puértolas-Balint F. Singh D. K. Wongkuna S. (2024). The gut commensal blautia maintains colonic mucus function under low-fiber consumption through secretion of short-chain fatty acids. Nat. Commun. 15, 3502. 10.1038/s41467-024-47594-w 38664378 PMC11045866

[B19] Hong J. Z. Fu Y. F. Chen X. Q. Zhang Y. Li X. Li T. (2024). Gut microbiome changes associated with chronic pancreatitis and pancreatic cancer: a systematic review and meta-analysis. Int. J. Surg. 110, 5781–5794. 10.1097/JS9.0000000000001724 38847785 PMC11392207

[B20] Jiang S. M. Zhao Y. J. Li Y. Y. Yang Q. (2020). Clinical research on progress of irritable bowel syndrome based on brain–gut axis. World Chin. Med. 15 (21), 3351–3358. 10.3969/j.issn.1673-7202.2020.21.030

[B21] Jiang H. Y. Ma R. A. Ji F. L. Liu Y. Wang B. Fu S. q. (2024). Structure characterization of polysaccharides from Cistanche deserticola and their neuroprotective effects against oxidative stress in slow transit constipation mice. Int. J. Biol. Macromol. 260 (Pt 2), 129527. 10.1016/j.ijbiomac.2024.129527 38246435

[B22] Kamalova A. Manoocheri K. Liu X. Casello S. M. Huang M. Baimel C. (2024). CCK+ interneurons contribute to thalamus-evoked feed-forward inhibition in the prelimbic prefrontal cortex. J. Neurosci. 44, e0957232024. 10.1523/JNEUROSCI.0957-23.2024 38697841 PMC11154858

[B23] Khan M. T. Zohair M. Khan A. Kashif A. Mumtaz S. Muskan F. (2025). From gut to brain: the roles of intestinal microbiota, immune system, and hormones in intestinal physiology and gut-brain-axis. Mol. Cell. Endocrinol. 607, 112599. 10.1016/j.mce.2025.112599 40482955

[B24] Lei J. Gong D. Duan L. Tang R. Gu W. Zhang F. (2025). A multidimensional perspective on poria cocos, an ancient fungal traditional Chinese medicine. J. Ethnopharmacol. 348, 119869. 10.1016/j.jep.2025.119869 40280371

[B25] Leng Y. Wei W. Tang X. D. (2025). Expert consensus on diagnosis and treatment of constipation by traditional Chinese medicine (2024). J. Traditional Chin. Med. 66, 321–328. 10.13288/j.11-2166/r.2025.03.019

[B26] Li X. X. Lu S. F. Zhu B. M. Jing X. Y. Fu S. P. (2019). Advances in research on serotonin and gut microbiota: their roles in gut-brain axis-associated disorders. Chin. J. Rehab. Med. 34 (1), 116–119. 10.3969/j.issn.1001-1242.2019.01.026

[B27] Li L. X. Feng X. Tao M. T. Paulsen B. S. Huang C. Feng B. (2022). Benefits of neutral polysaccharide from rhizomes of Polygonatum sibiricum to intestinal function of aged mice. Front. Nutr. 9, 992102. 10.3389/fnut.2022.992102 36204377 PMC9531825

[B28] Liang X. J. Chen L. L. Feng X. L. Liu J. L. Peng Y. M. (2025). Effect of massa medicata fermentata on gastrointestinal hormones and intestinal flora in mice with spleen deficiency constipation based on network pharmacology. Chin. J. New Drugs 34 (19), 2076–2086. 10.20251/j.cnki.1003-3734.2025.19.009

[B29] Lin F. Guo L. L. Wang J. (2014). Expounding the functions of Qi in TCM based on the effect of mitochondria. Chin. J. Integr. Tradit. West. Med. 34 (8), 903–906. 10.7661/CJIM.2014.08.0903 25223169

[B30] Liu Y. F. Lang H. Y. Yang L. M. Wang J. Zhang Y. Y. (2018). Modern interpretation of the correlation between the spleen controlling blood and the integrated functional effect of multiple zang-fu. China J. Traditional Chin. Med. Pharm. 33 (8), 3679–3681.

[B31] Liu X. L. Zhao H. H. Qin Z. P. Sun N. N. (2020). An analysis of Qi and modern research. Clin. J. Chin. Med. 12 (14), 23–25.

[B32] Liu D. Tang W. Han C. Nie S. (2022). Advances in Polygonatum sibiricum polysaccharides: extraction, purification, structure, biosynthesis, and bioactivity. Front. Nutr. 9, 1074671. 10.3389/fnut.2022.1074671 36545471 PMC9760828

[B33] Liu R. L. Zhang X. L. Cai Y. H. Xu S. Xu Q. Ling C. (2024). Research progress on medicinal components and pharmacological activities of Polygonatum sibiricum. J. Ethnopharmacol. 328, 118024. 10.1016/j.jep.2024.118024 38484952

[B34] Lu J. Tian J. Zhou Li Meng L. Chen S. Ma C. (2021). Phytochemistry and biological activities of poria. J. Chem. 20, 6659775. 10.1155/2021/6659775

[B35] Luo M. Xie P. Deng X. Fan J. Xiong L. (2025). Bifidobacterium lactobacillus triple viable alleviates slow transit constipation by regulating gut microbiota and metabolism. J. Gastroenterol. Hepatol. 40 (6), 1561–1573. 10.1111/jgh.16960 40183209 PMC12136808

[B36] Muro P. Zhang L. Li S. X. Zhao Z. Jin T. Mao F. (2024). The emerging role of oxidative stress in inflammatory bowel disease. Front. Endocrinol. (Lausanne) 15, 1390351. 10.3389/fendo.2024.1390351 39076514 PMC11284038

[B37] Niu M. L. Zhen H. H. Tang C. X. Liang W. T. (2023). Clinical effect of modified jichuanjian on senile patients with slow transit constipation of spleen-Kidney yang deficiency syndrome and effect on brain-gut peptide. Chin. J. Exp. Traditional Med. Formulae 29 (11), 126–132. 10.13422/j.cnki.syfjx.20221290

[B38] Quigley E. M. M. (2017). Microbiota-brain-gut axis and neurodegenerative diseases. Curr. Neurol. Neurosci. Rep. 17, 94. PMID: 29039142. 10.1007/s11910-017-0802-6 29039142

[B39] Reich N. Hölscher C. (2024). Cholecystokinin (CCK): a neuromodulator with therapeutic potential in alzheimers and parkinsons disease. Front. Neuroendocrinol. 73, 101122. 10.1016/j.yfrne.2024.101122 38346453

[B40] Ren Y. Sun Y. Liao Y. Y. Wang S. Liu Q. Duan C. Y. (2024). Mechanisms of action and applications of Polygonatum sibiricum polysaccharide at the intestinal mucosa barrier: a review. Front. Pharmacol. 19, 1421607. 10.3389/fphar.2024.1421607 PMC11366640 39224782

[B41] Salari N. Ghasemianrad M. Ammari-Allahyari M. Rasoulpoor S. Shohaimi S. Mohammadi M. (2023). Global prevalence of constipation in older adults: a systematic review and meta-analysis. Wien Klin. Wochenschr 135, 389–398. 10.1007/s00508-023-02156-w 36826591

[B42] Scott S. M. Simrén M. Farmer A. D. Dinning P. G. Carrington E. V. Benninga M. A. (2021). Chronic constipation in adults: contemporary perspectives and clinical challenges. 1: epidemiology, diagnosis, clinical associations, pathophysiology and investigation. Neurogastroenterol. Motil. 33, e14050. 10.1111/nmo.14050 33263938

[B43] Sharp T. Barnes N. M. (2020). Central 5-HT receptors and their function; present and future. Neuropharmacology 177, 108155. 10.1016/j.neuropharm.2020.108155 32522572

[B44] Shi L. L. Chen F. Wu Y. Z. Zhan C. R. Liu M. Tao L. Y. (2021). An experimental study on liqi tongbian mixtures regulation of brain-gut peptides and amelioration of visceral hypersensitivity in a rat model of constipation-predominant irritable bowel syndrome. Zhejiang J. Tradit. Chin. Med. 56 (8), 550–552. 10.13633/j.cnki.zjtcm.2021.08.003

[B45] Staller K. Olén O. Söderling J. Roelstraete B. Törnblom H. Song M. (2022). Chronic constipation as a risk factor for colorectal cancer: results from a nationwide, case-control study. Clin. Gastroenterol. Hepatol. 20 (8), 1867–1876.e2. 10.1016/j.cgh.2021.10.024 34687968 PMC9018894

[B46] Strandwitz P. (2018). Neurotransmitter modulation by the gut microbiota. Brain Res. 1693 (Pt B), 128–133. 10.1016/j.brainres.2018.03.015 29903615 PMC6005194

[B47] Tang S. H. Li S. H. Lin X. Y. Ke X. Ke M. H. Haung M. H. (2021). Effects of liqi tongbian formula on brain‐gut peptides in rats with functional constipation of qi stagnation pattern. J. Beijing Univ. Tradit. Chin. Med. 44 (7), 615–624. 10.3969/j.issn.1006-2157.2021.07.006

[B48] Tao Y. T. Xue Y. M. Qin P. Y. Zhang Z. Y. Mu J. K. Yang X. X. (2021). Analysis on the medication compatibility of ancient formulas containing polygonatum in successive dynasties. Chin. J. Ethnomed. Ethnopharm. 30 (6), 4–10.

[B49] Tsaturyan V. Poghosyan A. Toczyłowski M. Pepoyan A. (2022). Oxidative stress and host-bacteria interactions: *Escherichia coli* and salmonella Derby. Cells 11, 2989. 10.3390/cells11192989 36230950 PMC9564265

[B50] Wang L. F. Wu F. Hong Y. L. Shen L. Zhao L. Lin X. (2022). Research progress in the treatment of slow transit constipation by traditional Chinese medicine. J. Ethnopharmacol. 290, 115075. 10.1016/j.jep.2022.115075 35134487

[B51] Wang P. Wang F. Zuo X. H. (2024a). Effect of zengye decoction and liumo decoction on levels of serum 5-HT, motilin and vasoactive intestinal peptide in diabetic patients with constipation. Liaoning J. Tradit. Chin. Med. 51 (5), 131–135.

[B52] Wang C. Gu Y. Chu Q. Wang X. Ding Y. Qin X. (2024b). Gut microbiota and metabolites as predictors of biologics response in inflammatory bowel disease: a comprehensive systematic review. Microbiol. Res. 282, 127660. 10.1016/j.micres.2024.127660 38442454

[B53] Wen J. (2025). Effect of transcranial magnetic stimulation combined with umbilical moxibustion on constipation after cerebral infarction and its influence on serum 5‐HT level. Chin. J. Mod. Drug Appl. 19 11, 150–153.

[B54] Wierzbicka A. Mańkowska-Wierzbicka D. Mardas M. Stelmach-Mardas M. (2021). Role of probiotics in modulating human gut microbiota populations and activities in patients with colorectal Cancer-A systematic review of clinical trials. Nutrients 13, 1160. 10.3390/nu13041160 33915854 PMC8066620

[B55] Wu W. Wang J. P. Zhao J. D. Zhang Y. R. Hao H. S. Qiao Y. (2022). Effects of sijunzi decoction on 5-HT receptor, serum inflammation and brain—gut peptide in patients with irritable bowel syndrome Chin. Arch. Tradit. Chin. Med. 1–7.

[B56] Wu Y. Li C. R. Yi X. Yu Z. Z. Tan Z. J. (2023). Effect of Dendrobium officinale on the diversity of intestinalmicrobiota in mice with spleen deficiency constipation. Chin. J. Microecol. 35 (09), 1026–1031. 10.13381/j.cnki.cjm.202309006

[B57] Xiao J. D. Xie L. M. Zheng B. Ma W. J. Chen Y. Xie J. H. (2024). Polygonatum cyrtonema saponin supplementation ameliorated DSS-Induced intestinal barrier injury via targeting the pi3kaktmtor-mediated autophagymicrobiota axis. Food Biosci. 61 (61), 104727. 10.1016/j.fbio.2024.104727

[B58] Xie Y. K. Pan X. Y. Liang X. R. Zhai K. F. Yu Q. (2025). Research progress on structural characterization and bioactivities of poria cocos and ganoderma polysaccharides. Food and Med. Homol. 2, 9420040. 10.26599/FMH.2025.9420040

[B59] Yang J. Yang Y. (2025). Observation on the clinical effect of shenrong tongbian prescription on the elderly patients with chronic functional constipation of yang deficiency type and its influence on serum brain-gut peptide. China Pract. Med. 20, 24–28.

[B60] Yi Y. L. Li Y. Guo S. Yan H. Ma X. f. Tao W. w. (2022). Elucidation of the reinforcing spleen effect of jujube fruits based on metabolomics and intestinal flora analysis. Front. Cell. Infect. Microbiol. 12, 847828. 10.3389/fcimb.2022.847828 35402299 PMC8987507

[B61] Yi X. Zhou K. Jiang P. Deng N. Peng X. Tan Z. (2023a). Brain-bacteria-gut axis and oxidative stress mediated by intestinal mucosal microbiota might be an important mechanism for constipation in mice. Biotech. 13, 192. 10.1007/s13205-023-03580-5 PMC10185723 37205176

[B62] Yi X. Zhou K. Deng N. Cai Y. Peng X. Tan Z. (2023b). Simo decoction curing spleen deficiency constipation was associated with brain-bacteria-gut axis by intestinal mucosal microbiota. Front. Microbiol. 14, 1090302. 10.3389/fmicb.2023.1090302 36846756 PMC9947565

[B63] You Y. Luo L. You Y. Lin Y. Hu H. Chen Y. (2020). Shengmai yin formula modulates the gut microbiota of spleen-deficiency rats. Chin. Med. 15, 114. 10.1186/s13020-020-00394-y 33133231 PMC7594433

[B64] Zhang Y. Sun M. J. Duan Y. T. Haung J. J. Wang L. Yao L. (2024). Study of poria cocos polysaccharide on immune function and intestinal flora regulating of spleen deficiency rats. China J. Tradit. Chin. Med. Pharm. 39 (10), 5474–5480.

[B65] Zhang Y. S. Wang H. Sang Y. W. Liu M. Wang Q. Yang H. (2020). Gut microbiota in health and disease: advances and future prospects. MedComm 5, e70012. 10.1002/mco2.70012 PMC11577303 39568773

[B66] Zhang F. L. Tian J. J. Li J. T. Zhang T. Wang X. (2025). Akkermansia muciniphila alleviates metabolic disorders through gut microbiota-mediated tryptophan regulation. Amb. Express 15, 181. 10.1186/s13568-025-01986-3 41273480 PMC12748435

[B67] Zhu S. B. Liu J. X. Zhang C. W. Feng Q. H. (2022). Animal constipation models combining the disease and syndrome: a review. World Chin. Med. 17 (13), 1955–1958. 10.3969/j.issn.1673-7202.2022.13.026

[B68] Zou Y. Zheng X. B. Dai S. X. Ye Q. L. Dan W. (2009). Establishment of mice model of spleen deficiency constipation. Beijing J. Traditional Chin. Med. 28 (1): 60–62. 10.16025/j.1674-1307.2009.01.029

